# Associations between longitudinal changes in sleep disturbance and depressive and anxiety symptoms during the COVID‐19 virus pandemic among older women with and without breast cancer in the thinking and living with breast cancer study

**DOI:** 10.1002/cam4.4682

**Published:** 2022-03-22

**Authors:** Traci N. Bethea, Wanting Zhai, Xingtao Zhou, Tim A. Ahles, Jaeil Ahn, Harvey J. Cohen, Asma A. Dilawari, Deena M. A. Graham, Heather S. L. Jim, Brenna C. McDonald, Zev M. Nakamura, Sunita K. Patel, Kelly E. Rentscher, James Root, Andrew J. Saykin, Brent J. Small, Kathleen M. Van Dyk, Jeanne S. Mandelblatt, Judith E. Carroll

**Affiliations:** ^1^ Office of Minority Health and Health Disparities Research Georgetown Lombardi Comprehensive Cancer Center Washington District of Columbia USA; ^2^ Cancer Prevention and Control Program Georgetown Lombardi Comprehensive Cancer Center Washington District of Columbia USA; ^3^ Department of Psychiatry and Behavioral Sciences Memorial Sloan Kettering Cancer Center New York New York City USA; ^4^ Department of Biostatistics, Bioinformatics, and Biomathematics Georgetown University Washington District of Columbia USA; ^5^ Center for the Study of Aging and Human Development Duke University Medical Center Durham North Carolina USA; ^6^ Medstar Washington Hospital Center Washington District of Columbia USA; ^7^ John Theurer Cancer Center Hackensack University Medical Center Hackensack New Jersey USA; ^8^ Moffitt Cancer Center Tampa Florida USA; ^9^ Department of Radiology and Imaging Sciences Indiana University School of Medicine and Indiana University Melvin and Bren Simon Comprehensive Cancer Center Indianapolis Indiana USA; ^10^ Department of Psychiatry University of North Carolina‐Chapel Hill Chapel Hill North Carolina USA; ^11^ City of Hope National Medical Center Los Angeles California USA; ^12^ Cousins Center for Psychoneuroimmunology University of California, Los Angeles Los Angeles California USA; ^13^ Semel Institute for Neuroscience and Human Behavior, Department of Psychiatry & Biobehavioral Sciences University of California, Los Angeles Los Angeles California USA; ^14^ College of Behavioral and Community Sciences, School of Aging Studies University of South Florida Tampa Florida USA

**Keywords:** behavioral science, breast cancer, epidemiology, psychosocial studies

## Abstract

**Purpose:**

Several studies have reported sleep disturbances during the COVID‐19 virus pandemic. Little data exist about the impact of the pandemic on sleep and mental health among older women with breast cancer. We sought to examine whether women with and without breast cancer who experienced new sleep problems during the pandemic had worsening depression and anxiety.

**Methods:**

Breast cancer survivors aged ≥60 years with a history of nonmetastatic breast cancer (*n* = 242) and frequency‐matched noncancer controls (*n* = 158) active in a longitudinal cohort study completed a COVID‐19 virus pandemic survey from May to September 2020 (response rate 83%). Incident sleep disturbance was measured using the restless sleep item from the Center for Epidemiological Studies‐Depression Scale (CES‐D). CES‐D score (minus the sleep item) captured depressive symptoms; the State‐Anxiety subscale of the State Trait Anxiety Inventory measured anxiety symptoms. Multivariable linear regression models examined how the development of sleep disturbance affected changes in depressive or anxiety symptoms from the most recent prepandemic survey to the pandemic survey, controlling for covariates.

**Results:**

The prevalence of sleep disturbance during the pandemic was 22.3%, with incident sleep disturbance in 10% and 13.5% of survivors and controls, respectively. Depressive and anxiety symptoms significantly increased during the pandemic among women with incident sleep disturbance (vs. no disturbance) (*β* = 8.16, *p* < 0.01 and *β* = 6.14, *p* < 0.01, respectively), but there were no survivor‐control differences in the effect.

**Conclusion:**

Development of sleep disturbances during the COVID‐19 virus pandemic may negatively affect older women's mental health, but breast cancer survivors diagnosed with the nonmetastatic disease had similar experiences as women without cancer.

## Graphical abstract

In this longitudinal study, the development of sleep disturbances during the COVID‐19 virus pandemic may have negatively affected older women's mental health by increasing symptoms of depression and anxiety. Breast cancer survivors diagnosed with non‐metastatic disease had similar experiences during the pandemic as women of similar age without cancer.

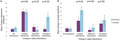

## 1 INTRODUCTION

The COVID‐19 virus pandemic and associated stress have been linked to altered sleep patterns^1^ and sleep disturbances,^2^, ^3^ including restless sleep and increased sleep duration.^4^, ^5^ In a meta‐analysis of cross‐sectional studies of adult men and women from 13 countries, the prevalence of sleep problems during the pandemic was as high as 32% in the general population.^2^

Sleep problems are associated with an increased risk for depression and anxiety.^6^, ^7^ Recent meta‐analyses of cross‐sectional studies estimate that the prevalence of depression has ranged from 17% to 32% and the prevalence of anxiety has ranged from 15% to 34% among various populations of adults during the pandemic.^8^, ^9^ To our knowledge, no studies have assessed whether the incidence of sleep disturbance during the pandemic is associated with increases in depressive and anxiety symptoms.

Breast cancer survivors tend to have poorer sleep than the general population,^10^, ^11^ often suffering sleep disturbances long after initial treatment.^12^, ^13^ Breast cancer survivors also experience greater depression and anxiety symptoms compared with women without cancer.^10^ The Utrecht cohort for Multiple Breast Cancer Intervention Studies and Long‐term Evaluation (UMBRELLA), a multisite study of breast cancer survivors aged 18 years and older, noted that depressive symptoms increased during the pandemic, though they did not find significant changes in anxiety or sleep disturbance and only examined age and breast cancer treatment as potential predictors of change in sleep disturbance.^14^

There are limited longitudinal data examining change in sleep from pre‐ to mid‐pandemic in older breast cancer survivors.^15^, ^16^ We conduct an analysis of data from the Thinking and Living with Cancer Study (TLC). TLC is a longitudinal cohort of breast cancer survivors aged 60 and older and matched noncancer controls who complete annual assessments over 60 months. We added an assessment to capture pandemic‐related well‐being and used these data to test relationships between changes in sleep and mental health from prior to the pandemic to mid‐pandemic. We hypothesized that women who developed a new sleep disturbance during the pandemic would have more depressive and anxiety symptoms during the pandemic compared with before the pandemic. Given the increased risk of adverse mental health outcomes among breast cancer survivors,^10^ we also postulated that the association between the development of sleep disturbances and changes in mental health would be greater for breast cancer survivors than noncancer controls. There is growing recognition of the importance of mental health among cancer survivors and the present study is intended to ascertain whether older breast cancer survivors are particularly vulnerable to sleep disturbance and mental health sequelae during the COVID‐19 virus pandemic.

## 2 METHODS

### 2.1 Study population

The TLC Study has been described in detail elsewhere.^17^ In brief, it is an ongoing multisite prospective cohort study of women aged ≥60 years that began recruitment in August 2010. Breast cancer survivors were women newly diagnosed with American Joint Committee on Cancer stage 0–III breast cancer^18^; controls were friends of survivors or community‐dwelling women frequency‐matched based on age, race, education, and region of residence. Exclusion criteria included the history of stroke, head injury, diagnosis of a major psychiatric or neurodegenerative disorder, treatment for a recent cancer diagnosis (within 5 years), and prior chemotherapy or hormonal therapy for cancer treatment. The baseline assessment included questionnaires, tests of cognitive functioning, and blood samples. Survivors completed their baseline visit before beginning systemic cancer treatment. Women complete assessments every 12 months for up to 60 months. Participation ends after the 60‐month follow‐up, at cancer recurrence or death, or study dropout. The study protocol (ClinicalTrials.gov: NCT03451383) was approved by Institutional Review Boards (IRBs) at each participating site (i.e., City of Hope Comprehensive Cancer Center [11255], Georgetown University [2008–363], Hackensack University Medical Center [Pro00005227], Indiana University School of Medicine [1602851718], Memorial Sloan Kettering Cancer Center [10‐079A], and Moffitt Cancer Center [MCC 16933]) and informed consent was obtained from all individual participants included in the study.

### 2.2 Data collection

**FIGURE 1:**
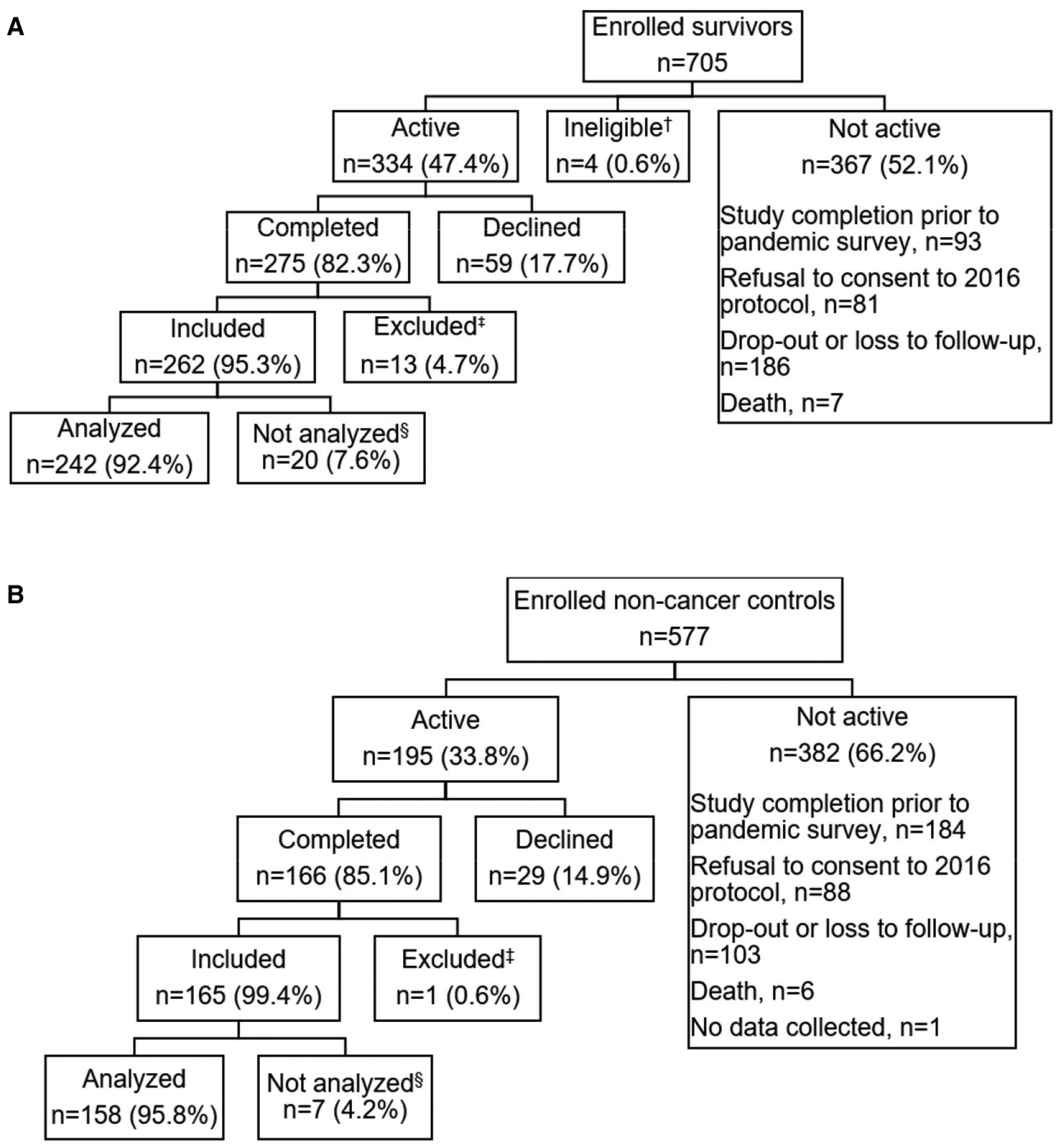
CONSORT diagram for the Thinking and Living with Cancer Study (Panel A: Survivors; Panel B: Noncancer controls); ^†^Survivors ineligible due to missing treatment information; ^‡^Participants excluded due to COVID‐19 virus diagnosis; ^§^Participants not included in analyses due to missing sleep data

We developed an IRB‐approved additional survey to evaluate the impact of the COVID‐19 virus pandemic on study measures. All active TLC participants were invited to respond to the pandemic survey. Reasons for not being active in the study included completing the study before 2016 (*n* = 277), when the study protocol added follow‐up and assessments; refusal to consent to the 2016 protocol (*n* = 169); death (*n* = 13); and study dropout or loss to follow‐up (*n* = 289). Active participants were similar to the remainder of the overall TLC sample in demographic and psychosocial characteristics, except for case status, region, and race (data not shown). Approximately 83% of active TLC participants (82.3% of survivors, 85.1% of controls) completed the pandemic survey (Figure 1), which was administered via telephone by study staff or self‐administered online between May 27, 2020 and September 11, 2020. Participants who reported a COVID‐19 virus diagnosis were excluded from analyses. We also excluded 27 participants without sleep data, resulting in a final analytic sample of 400 participants (Figure 1). Compared with respondents, nonrespondents were more likely to be non‐White and to report more depressive and anxiety symptoms at baseline (Table S1).

### 2.3 Exposure measures

One question from the Center for Epidemiological Studies‐Depression Scale (CES‐D)—“During the past week, my sleep was restless”—was used as a measure of sleep disturbance because we had these data on all women from all time points. Participants who reported restless sleep “occasionally or a moderate amount of time (3–4 days)” or “most or all of the time (5–7 days)” were considered to have sleep disturbance versus no/minimal sleep disturbance.

We used data from the most recently completed prepandemic survey and the COVID‐19 virus pandemic survey to characterize the change in sleep disturbance categorized as follows: (1) no disturbance (no sleep disturbance reported on both surveys), (2) incident disturbance (no sleep disturbance at the most recent prepandemic survey and sleep disturbance reported on the pandemic survey), (3) resolved disturbance (sleep disturbance at the most recent prepandemic survey and no sleep disturbance at the pandemic survey), and (4) persistent disturbance (sleep disturbance reported on both surveys).

In a secondary analysis, we assessed sleep duration using the question “During the past month, how many hours of actual sleep did you get at night?” from the Pittsburgh Sleep Quality Index.^19^ We used data from the most recently completed prepandemic survey and the pandemic survey to characterize the change in sleep duration, which was categorized as follows: ≥1 h decrease, no/negligible change, and ≥1 h increase. One hour was selected as the cutoff for change in sleep time due to interpretability. Findings did not markedly differ from alternative measures such as standard deviation or tertiles (data not shown).

### 2.4 Outcome measures

We used the 20‐item CES‐D scale^20^, ^21^ to measure depressive symptoms, with a higher CES‐D score reflecting more depressive symptoms. In analyses for change in sleep disturbance, the restless sleep item was removed from the CES‐D score. The internal consistency for the CES‐D was high with and without the restless sleep item (both Cronbach's α: 0.87). We used the 20‐item State‐Anxiety subscale of the State Trait Anxiety Inventory (STAI)^22^ as a measure of current anxiety. The STAI score ranges from 20 to 80, where a higher score reflects more anxiety. A score of 39–40 indicates clinically relevant anxiety symptoms in the general population, whereas scores of 44–55 have been used for older populations.^23^, ^24^

The CES‐D and STAI were included on annual follow‐up surveys and on the pandemic survey. Change in each measure was calculated as the difference between the score on the pandemic survey and the score on the most recent prepandemic survey. We defined a score of 10 as a clinically meaningful change in CES‐D^25^ and a score of 8 as a clinically meaningful change in STAI in accordance with previously published results.^26^

### 2.5 Covariates

Data on potential sociodemographic and health‐related covariates were collected from baseline, including age, race (White, non‐White), Wide Range Achievement Test, 4th edition Word Reading subtest (WRAT), time (in months) from the woman's most recent prepandemic survey to the pandemic survey, and county‐level COVID‐19 virus deaths in the woman’s area of residence. The rate of COVID‐19 virus deaths was calculated as the cumulative number of COVID‐19 virus deaths per 1000 individuals from initial data reporting in each U.S. county to the date that the woman completed her pandemic survey, divided by the 2019 county population estimate^27^ based on reports from state and local health agencies.^28^ County‐level COVID‐19 virus mortality was selected to estimate the local severity of the pandemic where each woman lived.

### 2.6 Statistical analyses

We described the distribution of measures and tested for differences between survivors and noncancer controls using means, standard deviations, and *t* tests for continuous variables and percentages and χ^2^ tests for categorical variables. Linear mixed‐effects regression models were used to examine the relation of change in sleep disturbance to change in CES‐D or STAI, controlling for age, survivor versus control, county‐level COVID‐19 virus mortality rate, and the months between participants' most recent prepandemic survey and the pandemic survey. We also considered the effect of adjusting for the CES‐D or STAI score on the most recent prepandemic questionnaire. In the secondary analysis, we examined the relation of change in sleep duration to change in depressive or anxiety symptoms with control for the same covariates. We then conducted an analysis with both change in sleep disturbance and change in sleep duration in the multivariable model. In analyses, we assessed interaction effects between each sleep measure and survivor versus control (case status) by including an interaction term in the multivariable model. Post hoc power calculations for the primary analyses indicated that we had ≥80% power to detect a small‐sized interaction (*d* = 0.28).^29^ Multiple testing correction was not applied and a two‐sided *p* < 0.05 was considered to be statistically significant. All analyses were conducted using SAS 9.4 (SAS Institute Inc.).

## 3 RESULTS

### 3.1 Study population

Women were an average of 67.9 years of age (range 60–88 years) and 85.8% identified as White with no differences in baseline factors between survivors and controls (Table 1). One‐quarter of participants reported sleep disturbance on the most recent prepandemic survey (Table 1). Survivors reported more depressive symptoms than controls (5.5 vs. 4.1, respectively, *p* = 0.03) prior to the pandemic, but anxiety scores were similar. There was an increase in sleep disturbances and depressive and anxiety symptoms during the pandemic, but survivors and controls did not differ in the magnitude of change (Table 1). Likewise, change in sleep duration was similar in survivors and controls (*p* = 0.53), with 20.9% of participants experiencing ≥1 h decrease in sleep duration, 24.1% experiencing ≥1 h increase in sleep duration, and 55% experiencing no or negligible change. On average, depressive and anxiety symptoms increased across all categories of change in sleep disturbance with the greatest increases in symptoms among participants who experienced incident disturbance, followed by women with persistent disturbances (Table S2).

**TABLE 1:** Distribution of baseline characteristics and sleep, depression, and anxiety measures among older women with breast cancer and noncancer controls^a^ ^,^ ^b^

Characteristic	Total	Controls	Survivors	*p* ^c^
	(*N* = 400)	(*N* = 158)	(*N* = 242)	
Baseline				
Age, mean ± SD (median)	67.9 ± 5.5 (67.0)	67.9 ± 5.8 (68.0)	67.9 ± 5.4 (67.0)	0.99
Marital status, *n* (%)				
Married	248 (62.3)	91 (57.6)	157 (65.4)	0.12
Widowed/divorced/single	150 (37.7)	67 (42.4)	83 (34.6)	
Region, *n* (%)				
Washington, D.C.	87 (21.8)	29 (18.4)	58 (24.0)	0.05
Indiana	113 (28.3)	56 (35.4)	57 (23.6)	
Los Angeles	16 (4.0)	3 (1.9)	13 (5.4)	
New York/New Jersey	71 (17.8)	25 (15.8)	46 (19.0)	
Tampa	113 (28.3)	45 (28.5)	68 (28.1)	
Race, *n* (%)				
White	343 (85.8)	140 (88.6)	203 (83.9)	0.19
Non‐White	57 (14.3)	18 (11.4)	39 (16.1)	
WRAT score, mean ± SD (median)	109.9 ± 15.12 (109.0)	110.8 ± 15.92 (109.0)	109.2 ± 14.58 (109.0)	0.30
Most recent prepandemic survey				
Sleep disturbance, *n* (%)				
None	296 (74.9)	124 (79.5)	172 (72.0)	0.09
Any	99 (25.1)	32 (20.5)	67 (28.0)	
Depressive symptoms, mean ± SD (median)	4.95 ± 6.44 (3.0)	4.06 ± 5.43 (2.0)	5.53 ± 5.97 (4.0)	0.03
Anxiety symptoms, mean ± SD (median)	27.76 ± 6.75 (25.0)	27.46 ± 7.12 (24.5)	27.95 ± 6.51 (26.0)	0.48
Pandemic survey				
Sleep disturbance, *n* (%)				
None	311 (77.8)	125 (79.1)	186 (76.9)	0.60
Any	89 (22.3)	33 (20.9)	56 (23.1)	
Depressive symptoms, mean ± SD (median)	7.68 ± 7.86 (5.0)	7.43 ± 8.12 (5.0)	7.85 ± 7.70 (5.0)	0.60
Anxiety symptoms, mean ± SD (median)	30.41 ± 9.45 (27.0)	30.73 ± 9.78 (27.0)	30.21 ± 9.24 (27.0)	0.59
Change between most recent prepandemic and pandemic surveys				
Change in sleep disturbance, n (%)				
No disturbance	251 (63.5)	103 (66.0)	148 (61.9)	0.24
Incident disturbance	45 (11.4)	21 (13.5)	24 (10.0)	
Resolved disturbance	55 (13.9)	20 (12.8)	35 (14.6)	
	44 (11.1)	12 (7.7)	32 (13.4)	
Change in depressive symptoms, mean ± SD (median)	2.76 ± 6.96 (1.0)	3.32 ± 6.84 (2.0)	2.39 ± 7.02 (1.0)	0.19
Change in anxiety symptoms, mean ± SD (median)	2.61 ± 8.28 (1.0)	3.15 ± 8.69 (1.0)	2.26 ± 8.00 (0.0)	0.30

^a^ Abbreviations: SD, standard deviation; WRAT, Wide Range Achievement Test.

^b^ The CES‐D score for depressive symptoms includes the restless sleep item.

^c^ Comparing survivors to noncancer controls.

Both survivors and controls with incident sleep disruption experienced a clinically meaningful increase in depressive symptoms (i.e., ≥10 points). However, among women with persistent sleep disturbance, depressive symptoms increased by 7.6 points in controls versus 1.3 points in survivors. For anxiety symptoms, among women with incident disturbance, controls experienced a clinically meaningful increase of 10.7 points, whereas survivors experienced a smaller increase of 5.3 points. Among women with persistent sleep disturbance, the increase in anxiety symptoms was similar in survivors and controls (4.03 and 5.36, respectively).

### 3.2 Associations between sleep disturbance and depressive and anxiety symptoms in the overall population

In multivariable regression models, relative to women who did not experience sleep disturbance, depressive symptoms significantly increased during the pandemic among women with incident disturbance (*β* = 8.2, *p* < 0.01), but not among those with resolved or persistent disturbance (Table 2). With adjustment for the most recent prepandemic CES‐D score, the association was similar for incident sleep disturbance (*β* = 8.7, *p* < 0.01) and stronger for persistent disturbance (*β* = 4.3, *p* < 0.01).

**TABLE 2:** Adjusted association of change in sleep disturbance with change in depressive symptoms and anxiety symptoms in older breast cancer survivors and noncancer controls

	Depressive symptoms^a^ ^,^ ^b^ ^,^ ^c^		Anxiety symptoms^a^ ^,^ ^b^ ^,^ ^d^	
	*β* (SE)	*p*	*β* (SE)	*p*
Change in sleep disturbance				
No disturbance	Reference		Reference	
Incident disturbance	8.16 (1.07)	<0.01	6.14 (1.31)	<0.01
Resolved disturbance	0.28 (0.98)	0.77	−0.31 (1.21)	0.80
Persistent disturbance	1.33 (1.08)	0.22	3.02 (1.35)	0.03
Age	0.05 (0.06)	0.42	−0.04 (0.08)	0.65
Case status				
Control	Reference		Reference	
Survivor	−0.67 (0.68)	0.32	−0.86 (0.84)	0.31
County‐level COVID‐19 virus mortality rate	0.29 (0.43)	0.50	−0.31 (0.52)	0.55
Months between surveys	−0.002 (0.07)	0.98	−0.10 (0.09)	0.23

^a^ Abbreviation: SE, standard error.

^b^ Model adjusts for all listed variables.

^c^ Model uses the CES‐D score without the restless sleep item and excludes 5 participants missing data on sleep disturbance and 10 participants missing data on depressive symptoms.

^d^ Model excludes 5 participants missing data on sleep disturbance and 3 participants missing data on anxiety symptoms.

In multivariable regression models, anxiety symptoms significantly increased from the prepandemic survey to the pandemic survey for women with an incident sleep disturbance and women with persistent sleep disturbance (*β* = 6.1, *p* < 0.01 and *β* = 3.0, *p* = 0.03, respectively), compared with no sleep disturbances (Table 2). With adjustment for the most recent prepandemic STAI score, the association was similar for incident sleep disturbance (*β* = 6.8, *p* < 0.01) and stronger for persistent disturbance (*β* = 5.3, *p* < 0.01).

The associations were similar with adjustment for change in sleep duration. For example, for incident disturbance, the parameter estimate was 7.98 (*p* < 0.01) for depressive symptoms and 5.6 (*p* < 0.01) for anxiety symptoms.

### 3.3 Associations between sleep disturbance and depressive and anxiety symptoms among breast cancer survivors and controls

**FIGURE 2:**
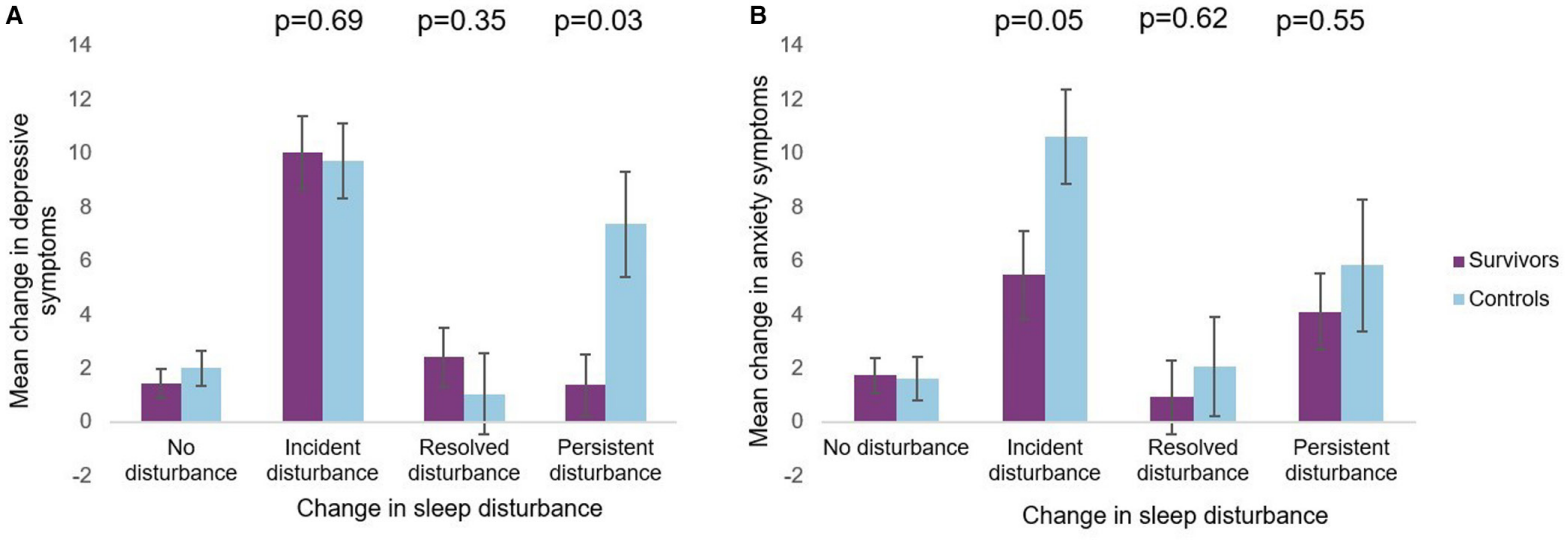
Change in sleep disturbance and adjusted mean change in depressive symptoms and anxiety symptoms, stratified by case status (Panel A: Depressive symptoms; Panel B: Anxiety symptoms; purple = survivors, blue = controls)

For depressive symptoms, there was no survivor‐control difference in the association with incident disturbance. However, an interaction between sleep disturbance and case status was present for persistent disturbance (p_interaction_ = 0.03, Figure 2A): the mean change in depressive symptoms was larger among controls than among survivors who had a persistent sleep disturbance (7.4 and 1.4, respectively). For anxiety symptoms, there was a marginally significant interaction between sleep disturbance and case status for incident disturbance (p_interaction_ = 0.05, Figure 2B): the mean change in anxiety symptoms was 5.5 among survivors and 10.6 among controls. There was no significant interaction between persistent disturbance and case status.

### 3.4 Associations between sleep duration and depressive and anxiety symptoms in the overall population and among breast cancer survivors and controls

**FIGURE 3:**
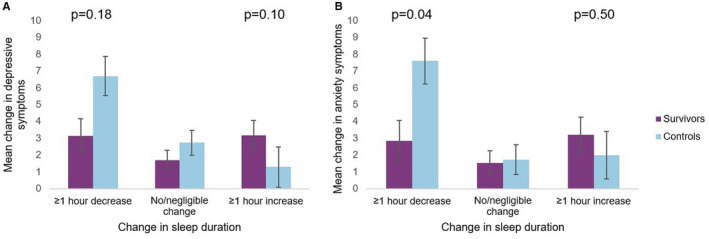
Change in sleep duration and adjusted mean change in depressive symptoms and anxiety symptoms, stratified by case status (Panel A: Depressive symptoms; Panel B: Anxiety; purple = survivors, blue = controls)

In the secondary analysis, depressive symptoms and anxiety symptoms significantly increased among participants who experienced ≥1 h decrease in sleep duration (*β* = 2.6, *p* < 0.01 and *β* = 3.3, *p* < 0.01, respectively), relative to participants who did not experience a change in sleep duration (Table 3). The results were attenuated with adjustment for change in sleep disturbance such that the association of a ≥1 h decrease in sleep duration on depressive symptoms and anxiety symptoms was no longer different from that of the reference group (*β* = 0.61, *p* = 0.5 and *β* = 1.71, *p* = 0.1, respectively). An interaction between change in sleep duration and case status was present for anxiety symptoms and decreasing sleep duration (p_interaction_ = 0.04, Figure 3B): the mean change in anxiety symptoms was 2.85 among survivors and 7.60 among controls.

**TABLE 3:** Adjusted association of change in sleep duration with change in depressive symptoms and anxiety symptoms in older breast cancer survivors and noncancer controls

	Depressive symptoms^a^ ^,^ ^b^ ^,^ ^c^		Anxiety symptoms^a^ ^,^ ^b^ ^,^ ^d^	
	*β* (SE)	*p*	*β* (SE)	*p*
Change in sleep duration				
≥1 h decrease	2.57 (0.91)	<0.01	3.28 (1.07)	<0.01
No/negligible change	Reference		Reference	
≥1 h increase	0.45 (0.86)	0.60	1.21 (1.02)	0.23
Age	0.03 (0.07)	0.66	−0.07 (0.08)	0.39
Case status				
Control	Reference		Reference	
Survivor	−0.89 (0.72)	0.22	−0.85 (0.85)	0.32
County‐level COVID‐19 virus mortality rate	0.63 (0.45)	0.16	−0.09 (0.52)	0.87
Months between surveys	−0.02 (0.07)	0.77	−0.11 (0.09)	0.19

^a^ Abbreviation: SE, standard error.

^b^ Model adjusts for all listed variables.

^c^ Model excludes 2 participants missing data on sleep duration and 10 participants missing data on depressive symptoms.

^d^ Model excludes 2 participants missing data on sleep duration and 3 participants missing data on anxiety symptoms.

## 4 DISCUSSION

This study is unique in studying the associations between incident sleep disturbance and depressive and anxiety symptoms during the COVID‐19 virus pandemic in the context of a longitudinal cohort study of older breast cancer survivors and a matched noncancer control group. We found that sleep disturbances were common during the pandemic and half of these were new sleep disturbances. We also observed clinically meaningful increases in depressive and anxiety symptoms from before the pandemic to during the pandemic period. Having an incident sleep disturbance was associated with larger increases in depressive and anxiety symptoms than having a resolved or persistent sleep disturbance, relative to not experiencing any sleep disturbance. However, contrary to expectations, the relationship of sleep disturbance with depressive and anxiety symptoms was not stronger among breast cancer survivors than among noncancer controls.

Survivors had more depressive symptoms prior to the pandemic, so controls had greater opportunities to increase CES‐D scores in response to sleep disturbance. In contrast, survivors and controls did not differ in anxiety symptoms before the pandemic. It is possible that the COVID‐19 virus pandemic, in addition to sleep disturbance, may represent a relatively larger source of anxiety for controls compared with survivors who are experiencing the pandemic within the context of other persistent stressors related to cancer diagnosis and treatment. Notably, controlling for the prepandemic CES‐D or STAI score strengthened associations with persistent sleep disturbance but did not affect results for incident sleep disturbance.

In a recent review, longitudinal studies of older adults with data before and during the pandemic have reported mixed findings regarding changes in depressive and anxiety symptoms.^30^ In a longitudinal study of Swedish adults before the pandemic, mean CES‐D scores increased by 1.6 points during 6 years of follow‐up.^31^ In a cross‐sectional study of cancer survivors, CES‐D scores, as well as mean scores for anxiety, remained consistent at around 10 points across ages 38 and 80.^32^ Based on these studies, the magnitude of change observed in TLC may be greater than expected during the follow‐up time that accrued between the most recent prepandemic survey and the pandemic survey. Our findings differ from those of the UMBRELLA study, a multisite study of breast cancer survivors aged 18 years and older, which found a significant increase in depressive symptoms but no mean difference in anxiety or insomnia symptoms comparing data collected before the pandemic with data collected during the pandemic.^14^ We report increases in both depressive and anxiety symptoms in breast cancer survivors and controls and demonstrate an association of sleep disturbances with these symptoms. The UMBRELLA study on breast cancer survivors during the COVID‐19 virus pandemic was descriptive, did not assess sleep as a predictor of changes in depressive or anxiety symptoms, and did not include a well‐matched control group. Potential differences in reported anxiety symptoms during the pandemic could be due to differences in the two cohorts, including differing country of residence, age range, and assessment measures. In particular, the inclusion of a control group in TLC enabled comparisons between breast cancer survivors.

To our knowledge, no studies on changes in sleep duration and mental health outcomes among cancer survivors during the COVID‐19 virus pandemic have been published to date. The results of our secondary analysis concur with large studies among the general adult population that observed both increases^33^, ^34^, ^35^ and decreases^33^ in sleep duration and studies that observed significant associations with depression and anxiety for decreasing sleep duration.^33^ However, the present study contrasts findings of significant associations with depression and anxiety for increasing sleep duration.^33^, ^36^

There are several potential explanations for our findings. Older adults are particularly vulnerable to deleterious COVID‐19 virus outcomes,^37^ including hospitalization and mortality. Awareness of this vulnerability could increase anxiety during the pandemic.^36^ Sleep disturbance may be contributing to the impact of the COVID‐19 virus pandemic on the health of older adults, with the potential for sleep disturbances to alter viral immune defenses,^38^ including reduced immunity after vaccination.^39^ Notably, some studies have proposed the use of melatonin, a hormone vital to the regulation of sleep–wake cycles and circadian rhythm, to promote physical health during the pandemic.^37^ Mindfulness meditation and cognitive behavioral therapy for insomnia have also been shown to be effective for addressing sleep disturbance.^40^, ^41^ These techniques may also prove to be beneficial for mental health, given that sleep impacts brain function, affect, and emotion regulation.^42^ Indeed, initial trials have demonstrated improvements in sleep and depressive symptoms using mindfulness meditation^41^ and cognitive behavioral therapy for insomnia,^43^ and several mind–body interventions have benefitted immune health by reducing inflammatory biology and improving viral immune defenses.^44^

This research must be considered in light of limitations. First, the prevalence of sleep disturbance was low in this older population. Estimates of the prevalence of sleep disturbance during the pandemic are as high as 78% among cancer survivors,^45^ though the prevalence likely differs by cancer type, disease severity, and recency of treatment. Women in TLC are well educated and tend to have ample social support, which could reduce pandemic‐related stress and sleep disturbance. In addition, TLC participants who did not respond to the pandemic survey were more likely to be non‐White and to report depressive and/or anxiety symptoms and older adults may be less likely to report depressive and anxiety symptoms.^46^, ^47^ Thus, our findings may not be generalizable to other populations of cancer survivors, populations of breast cancer survivors that differ in age or race/ethnicity, and survivors living with metastatic breast cancer. During the course of the pandemic, infection and mortality rates have ebbed and flowed with multiple waves such that our findings may have differed if data were collected at a different time point in the trajectory of the pandemic. Women's experiences of depressive and anxiety symptoms could be affected by their (in)ability to access medical care during the pandemic. Although depressive symptoms were weakly associated with disruptions in medical care among survivors in TLC, clinical factors were not associated with disruptions in medical care.^48^ As a result, it is unlikely that difficulties accessing healthcare strongly influenced the results of the present study. These data cannot differentiate between changes in sleep disturbances and in depressive and anxiety symptoms that occurred simultaneously and those that occurred sequentially. Thus, the results observed may not represent causal associations. Finally, depressive and anxiety symptoms were both assessed as primary outcomes and the statistically significant interactions observed may not remain with correction for multiple testing.

Our study benefits from longitudinal data collection prior to and during the pandemic and consideration of the observed changes in depressive and anxiety symptoms in relation to cutoffs for clinically meaningful change. Since our measure of depressive symptoms removed the restless sleep item, our results are likely conservative. Sleep disturbance and depression have a bidirectional relationship, so our focus on change in sleep in relation to changes in depression helps the analyses be less vulnerable to reverse causation than a study without prepandemic data or an analysis that relies on cross‐sectional data. Last, our study addresses the need for research on sleep disturbance and duration among older adults and cancer survivors. Sleep is a vital contributor to mental health and well‐being and the potential impact of the COVID‐19 virus pandemic on older adults and cancer survivors has been understudied.^15^, ^16^, ^37^, ^49^ Nevertheless, additional studies are needed to confirm our findings. Future investigation in TLC will examine whether changes in sleep that took place during the pandemic are maintained and whether there are long‐term health effects related to these changes.

This report is unique in being able to test the relationship of sleep disturbances with mental health changes during the pandemic in older women participating in a longitudinal cohort study. Our findings suggest that clinical and public health programs serving older adults should incorporate surveillance of sleep disturbance during the COVID‐19 virus pandemic in order to intercept worsening mental health. In our study, breast cancer survivors were not more vulnerable to the mental health outcomes associated with sleep disturbance, but many survivors receive care from oncology practices for years after their diagnosis. Thus, this work supports recent recommendations to integrate sleep health with oncology practice and related clinical care.^50^, ^51^, ^52^

## 5 CONCLUSIONS

In this study of older women with and without breast cancer, changes in sleep during the COVID‐19 virus pandemic were associated with increases in depressive and anxiety symptoms, and women with incident sleep disturbance experienced the greatest increases in symptoms, which were clinically meaningful changes. However, there was no evidence that survivors diagnosed with nonmetastatic breast cancer were at increased risk compared with women without cancer.

## CONFLICT OF INTEREST

Author Dilawari served on the Oncology Summit Advisory Board for Cardinal Health. Author Jim has consulted for RedHill BioPharma, Janssen Scientific Affairs, and Merck and has received grant funding from Kite Pharma. All other authors declare no conflicts.

## AUTHOR CONTRIBUTIONS

Conceptualization: TNB, JSM, and JEC; Formal analysis: TNB, WZ, and BJS; Data curation: XZ; Writing—original draft: TNB; Writing—review and editing: WZ, XZ, TAA, JA, HJC, AAD, DMG, HSJ, BCM, ZMN, SKP, KER, JR, AJS, BJS, KMV, JSM, and JEC; Supervision: JSM and JEC; Funding acquisition: TAA, JR, AJS, and JSM. All authors read and approved the final manuscript.

## ETHICS STATEMENT

All procedures performed in this study were in accordance with the ethical standards of the Institutional Review Boards for each participating site (i.e., City of Hope Comprehensive Cancer Center [11255], Georgetown University [2008‐363], Hackensack University Medical Center [Pro00005227], Indiana University School of Medicine [1602851718], Memorial Sloan Kettering Cancer Center [10‐079A], and Moffitt Cancer Center [MCC 16933]), the U.S. Federal Policy for the Protection of Human Subjects, and with the 1964 Declaration of Helsinki and its later amendments. Informed consent was obtained from all individual participants included in the study.

## CLINICAL TRIAL REGISTRATION

ClinicalTrials.gov: NCT03451383

## Supporting information


Table 1
Table 2

## Data Availability

The data that support the findings of this study are available on request from the senior authors. The data are not publicly available due to privacy or ethical restrictions.
