# Supplementary material for: Associations between longitudinal changes in sleep disturbance and depressive and anxiety symptoms during the COVID‐19 virus pandemic among older women with and without breast cancer in the thinking and living with breast cancer study
Source: Cancer Med. 2022 Mar 22;11(17):3352–63. doi: 10.1002/cam4.4682 (PMC9110906; doi:10.1002/cam4.4682)
Supplement: Supplementary file 1 — Table 1 Table 2 [file CAM4-11-3352-s001.docx]

**Supporting Information**

Supplemental Table 1. Distribution of baseline characteristics and pre-pandemic sleep, depression, and anxiety measures among older women with breast cancer and non-cancer controls by response to the TLC pandemic survey

| **Characteristic^†^** | **Non-respondents** |  | **Respondents** |  | ***P^‡^*** |
| --- | --- | --- | --- | --- | --- |
|  | **(N=88)** |  | **(N=427)** |  |  |
| *Baseline* |  |  |  |  |  |
| Age, mean ± sd. (median) | 68.0 ± 6.0 (66.5) |  | 67.9 ± 5.6 (67) |  | 0.91 |
| Case status |  |  |  |  | 0.32 |
| Control | 29 (33.0) |  | 165 (38.6) |  |  |
| Survivor | 59 (67.0) |  | 262 (61.4) |  |  |
| Region |  |  |  |  | 0.02 |
| Washington, D.C. | 22 (25.0) |  | 89 (20.8) |  |  |
| Indiana | 26 (29.5) |  | 113 (26.5) |  |  |
| Los Angeles | 5 (5.7) |  | 23 (5.4) |  |  |
| New York/New Jersey | 26 (29.5) |  | 88 (20.6) |  |  |
| Tampa | 9 (10.2) |  | 114 (26.7) |  |  |
| Race |  |  |  |  | <0.01 |
| White | 64 (72.7) |  | 368 (86.2) |  |  |
| Non-White | 24 (27.3) |  | 59 (13.8) |  |  |
| WRAT score, mean ± sd. (median) | 109.4 ± 15.0 (107.0) |  | 110.0 ± 15.1 (109.0) |  | 0.71 |
|  |  |  |  |  |  |
| *Most recent pre-pandemic survey* |  |  |  |  |  |
| Sleep disturbance |  |  |  |  | 0.15 |
| None | 53 (67.1) |  | 296 (74.9) |  |  |
| Any | 26 (32.9) |  | 99 (25.1) |  |  |
| Depressive symptoms (CES-D) , mean ± sd. (median) | 6.6 ± 7.8 (3.5) |  | 5.0 ± 6.4 (3.0) |  | 0.05 |
| Anxiety symptoms (STAI) , mean ± sd. (median) | 29.5 ± 9.5 (26.0) |  | 27.8 ± 6.8 (25.0) |  | 0.05 |
|  |  |  |  |  |  |

^†^Abbreviations: CES-D, Center for Epidemiological Studies-Depression Scale; sd, standard deviation; STAI, State-Trait Anxiety Inventory; WRAT, Wide Range Achievement Test

^‡^Comparing non-respondents to respondents

Supplemental Table 2. Distribution of change in depressive symptoms and anxiety symptoms from before the COVID-19 virus pandemic to during the pandemic within categories of change in sleep disturbance among older women with breast cancer and non-cancer controls, overall and by case status

|  | **Change in sleep disturbance^†,‡^** | | | | | | | | |  | |
| --- | --- | --- | --- | --- | --- | --- | --- | --- | --- | --- | --- |
|  | **No disturbance** | | **Incident disturbance** | | **Resolved disturbance** | | **Persistent disturbance** | |  | |  |
|  | **N** | **Mean (SD)** | **N** | **Mean (SD)** | **N** | **Mean (SD)** | **N** | **Mean (SD)** | ***P^§^*** | |  |
| ***Mean change in depressive symptoms*** |  |  |  |  |  |  |  |  |  | |  |
| Total | 248 | 1.67 (5.09) | 44 | 9.93 (10.20) | 54 | 1.98 (6.41) | 43 | 2.93 (8.55) |  | |  |
| Controls | 103 | 1.95 (4.86) | 21 | 9.71 (10.47) | 19 | 1.26 (5.69) | 11 | 7.55 (8.05) | 0.06 | |  |
| Survivors | 145 | 1.46 (5.26) | 23 | 10.13 (10.18) | 35 | 2.37 (6.82) | 32 | 1.34 (8.25) |  |  |  |
|  |  |  |  |  |  |  |  |  |  | |  |
| ***Mean change in anxiety symptoms*** |  |  |  |  |  |  |  |  |  | |  |
| Total | 250 | 1.74 (6.58) | 45 | 7.82 (13.50) | 55 | 1.35 (7.89) | 43 | 4.37 (8.72) |  | |  |
| Controls | 103 | 1.69 (6.52) | 21 | 10.71 (14.14) | 20 | 1.85 (7.59) | 11 | 5.36 (8.43) | 0.22 | |  |
| Survivors | 147 | 1.77 (6.64) | 24 | 5.29 (12.67) | 35 | 1.06 (8.15) | 32 | 4.03 (8.92) |  |  |  |
|  |  |  |  |  |  |  |  |  |  | |  |

^†^Abbreviations: sd, standard deviation

^‡^10 participants are missing data on depressive symptoms; 3 participants are missing data on anxiety symptoms; 5 participants are missing data on restless sleep

^§^Comparing survivors to non-cancer controls
